# Gene co-expression networks in liver and muscle transcriptome reveal sex-specific gene expression in lambs fed with a mix of essential oils

**DOI:** 10.1186/s12864-018-4632-y

**Published:** 2018-04-04

**Authors:** Marcella Sabino, Victor Adriano Okstoft Carmelo, Gianluca Mazzoni, Katia Cappelli, Stefano Capomaccio, Paolo Ajmone-Marsan, Andrea Verini-Supplizi, Massimo Trabalza-Marinucci, Haja N. Kadarmideen

**Affiliations:** 1 Dipartimento di Medicina Veterinaria, University of Perugia, Perugia, Italy; 2 Department of Bio and Health Informatics, Technical University of Denmark, Kongens Lyngby, Copenhagen, Denmark; 3 Istituto di Zootecnica, Catholic University of the Sacred Heart, Piacenza, Italy

**Keywords:** Essential oils, Lamb, Liver, Muscle, RNA-Seq, WGCNA

## Abstract

**Background:**

Essential oil (EO) dietary supplementation is a new strategy to improve animal health. EO compounds have antiparasitic, antimicrobial, antiviral, antimycotic, antioxidant and anti-inflammatory proprieties. Nutrigenomics investigations represent innovative approaches in understanding the relation between diet effect and gene expression related to the animal performance. Few nutrigenomics studies have used a high-throughput RNA-Sequencing (RNA-Seq) approach, despite great potential of RNA-Seq data in gene expression quantification and in co-expression network analyses. Our aim is to use the potential of RNA-Sequencing data in order to evaluate the effect of an EO supplementary diet on gene expression in both lamb liver and muscle.

**Results:**

Using a treatment and sex interaction model, 13 and 4 differentially expressed genes were identified in liver and muscle respectively. Sex-specific differentially expressed (DE) genes were identified in both sexes. Using network based analysis, different clusters of co-expressed genes that were highly correlated to the diet were detected in males vs. females, in agreement with DE analysis. A total of five regulatory genes in liver tissue associated to EO diet were identified: *DNAJB9, MANF, UFM1, CTNNLA1* and *NFX1*. Our study reveals a sex-dependent effect of EO diet in both tissues, and an influence on the expression of genes mainly involved in immune, inflammatory and stress pathway.

**Conclusion:**

Our analysis suggests a sex-dependent effect of the EO dietary supplementation on the expression profile of both liver and muscle tissues. We hypothesize that the presence of EOs could have beneficial effects on wellness of male lamb and further analyses are needed to understand the biological mechanisms behind the different effect of EO metabolites based on sex. Using lamb as a model for nutrigenomics studies, it could be interesting to investigate the effects of EO diets in other species and in humans.

**Electronic supplementary material:**

The online version of this article (10.1186/s12864-018-4632-y) contains supplementary material, which is available to authorized users.

## Background

In recent years, an increased attention has been given to improve animal performance and welfare improvements, using alternative strategies which do not include the use of synthetic molecules, and plant extracts are increasingly used as dietary supplements to pursue this objective [1–7].

Amongst plant extracts, essential oils have been used in ruminants for a number of different purposes. They can show antiparasitic, antimicrobial, antiviral, antimycotic, antioxidant and anti-inflammatory properties [8–11].

Oxidative stability and sensory quality of meat from lambs fed for 80 days a rosemary extract rich in terpenes (carnosic acid and carnosol) was found to be improved [12]. In another study, dietary oregano essential oil was shown to delay lipid oxidation of lamb meat [13].

As for the antiparasitic activity, amongst the others, thymus essential oil acts against the ovine gastrointestinal parasite *Haemonchus contortus* [14], while eucalyptus essential oil possesses anthelmintic activity against gastrointestinal nematodes [15]. Most essential oils have been used for their antimicrobial and antimycotic properties. For instance, dill essential oil is effective against *Staphylococcus*, *Streptococcus, Escherichia coli* and *Pseudomonas* [10], besides being able to induce *Candida albicans* apoptosis [16]. It also contains phenolic compounds with antioxidant and anti-inflammatory activities [11]. Cinnamon essential oil exhibits an activity in vitro against *Candida* spp. [17] and *Aspergillus* [18], while improving rumen fermentation in calves [19].

In several in vitro and in vivo studies, extracts from rosemary, oregano, dill, cinnamon, eucalyptus, garlic, clove or thymus were able to modulate ruminal methane emission to various extent, especially by acting on Gram+ bacteria [20]. Cobellis et al. (2016) [21] demonstrated that a 1:1:1 combination of dill seed, eucalyptus leaves and cinnamon bark essential oils can inhibit in vitro methane and ammonia production by 52% and 49%, respectively, without affecting ruminal dry matter degradability to a great extent (− 4%). In addition to livestock studies, a number of researches describe the protective and beneficial effects of essential oils in different tissues in humans. Indeed, it has been reported that pine, lime and eucalyptus essential oils possess antioxidant proprieties and act in reducing reactive oxygen species (ROS) in LPS-activated alveolar macrophages isolated from chronic pulmonary disease patients [22]. Thyme essential oil possesses anti cancer proprieties and act in reducing proliferation of oral carcinoma cells [23], while cinnamon essential oil exhibits anti tumor effects in HEp-2 cells by suppressing the expression of epidermal growth factor receptor-tyrosine kinase.

Tools for molecular data production and analysis are now available to investigate the link between nutrients and their effects on metabolism. Nutrigenomics is one of the innovative approaches that can shed light on the relationship between diet, gene expression and modulation of biological processes [24]. To date, qRT-PCR and/or microarray technologies have been extensively used to evaluate gene expression in nutrigenomics studies [25–28], but compared to these technologies, a high-throughput RNA-Sequencing approach (RNA-Seq) provides more accurate quantification of gene expression [29] and a view of the whole transcriptome [30–32]. RNA-Seq data is now widely used in differentially expressed genes identification, and it has also been successfully applied in gene co-expression network analysis [29, 32]. Co-expressed genes may be involved in similar pathways [33] and network analysis, through the use of Weighted Gene Correlation Network Analysis R package WGCNA [34], allows the identification of important regulatory genes that are good candidates to influence phenotypic traits of interest. This approach was successfully used to identify biomarkers for intestinal parasite resistance in sheep [35], however, despite this great potential, RNA-Seq data and co-expression network analyses have not been applied to lamb nutrigenomics studies.

Here we propose a nutrigenomics study aimed at evaluating the effect of a cinnamon bark, dill seed and eucalyptus leaves essential oil mix on the liver and muscle transcriptome profile in lamb. This mix has previously shown to be effective in modulating methane ruminal production [21] and meat oxidative stability (Trabalza-Marinucci, unpublished data). We used RNA-Seq data for detecting differentially expressed genes between two lamb groups, fed either a control diet or an essential oil mix supplemented diet. Furthermore, we used WGCNA to identify modules of co-expressed genes highly correlated to the dietary treatment.

## Results

### RNA sequencing

A total of 32 RNA samples, 16 for muscle and for liver, respectively, were sequenced and analysed using an optimised pipeline summed up in the workflow (Fig. 2). The RNA sequencing experiment produced an average of 14 million and 36 million read pairs in liver and muscle, respectively. Quality control and trimming procedures preserved 96.73% of reads in liver and 87.83% in muscle. Also, in both tissues 97% of cleaned reads were aligned successfully, and 82.49% of reads were uniquely mapped in liver, and 81.67% in muscle. Only uniquely mapped reads were used for the gene expression analysis.

### Differential gene expression analysis

#### Liver

In the differential expression analysis of the liver tissue thirteen significant genes were identified for the interaction between sex and diet (interaction genes) using a data set with 11.773 genes in DESEQ2. Fifteen genes were DE between EO and CTRL in males. Among these, fourteen were down regulated in EO [fibrinogen beta chain (*FGB*); fibrinogen gamma chain (*FGG***)**, DnaJ heat shock protein family (Hsp40) member B9 (*DNAJB9),* heat shock protein 90 beta family member 1 (*HSP90B1),* proteasome subunit alpha 4 *(PSMA4),* KDEL endoplasmic reticulum protein retention receptor 2 (*KDELR2),* Sjogren syndrome antigen B *(SSB),* calmodulin 2 (*CALM2),* signal peptidase complex subunit 3 (*SPCS3),* ubiquitin fold modifier 1 (*UFM1),* mitochondrial ribosomal protein S18B *(MRPS18B),* nucleolar protein interacting with the FHA domain of MKI67 (*NIFK),* arginase 1 (*ARG1),* transmembrane p24 trafficking protein 7 (*TMED7*)], while cytochrome P450 2D14 (*CYP2D6)* was up regulated in EO (Table 1).

**Table 1:** Interaction genes and DE genes in male in liver tissue (p-adj < 0.05)

Symbol	Gene description	L2FC	p-adj	
*CALM2*	calmodulin	-0,97	1,28E-02	Interaction
*DNAJB9*	DnaJ heat shock protein family (Hsp40) member B9	− 1,83	1,66E-03	Interaction
*FGB*	fibrinogen beta chain	−1,4	3,80E-03	Interaction
*FGG*	fibrinogen gamma chain	−1,28	1,66E-03	Interaction
*HERC2*	E3 ubiquitin-protein ligase	0,83	4,96E-03	Interaction
*HP*	haptoglobin	−1,54	4,03E-02	Interaction
*HSP90B1*	heat shock protein 90 beta family member 1	−1,43	1,28E-02	Interaction
*KDELR2*	KDEL endoplasmic reticulum protein retention receptor 2	-1,06	1,28E-02	Interaction
*MANF*	mesencephalic astrocyte derived neurotrophic factor	−2,23	3,71E-02	Interaction
*MRPS18B*	mitochondrial ribosomal protein S18B	−1,22	4,96E-03	Interaction
*NIFK*	nucleolar protein interacting with the FHA domain of MKI67	−1,29	3,71E-02	Interaction
*TMEM45A*	transmembrane protein 45A	−3,09	1,66E-03	Interaction
*UFM1*	ubiquitin fold modifier 1	−1,66	4,54E − 03	Interaction
*ARG1*	arginase 1	−0,74	4,85E-03	Male
*CALM2*	calmodulin	-0,64	3,98E-02	Male
*DNAJB9*	DnaJ heat shock protein family (Hsp40) member B9	−1,2	4,45E − 03	Male
*FGB*	fibrinogen beta chain	−0,97	4,45E − 03	Male
*FGG*	fibrinogen gamma chain	−0,91	4,21E − 03	Male
*HSP90B1*	heat shock protein 90 beta family member 1	−0,99	1,57E − 02	Male
*KDELR2*	KDEL endoplasmic reticulum protein retention receptor 2	-0,74	1,58E-02	Male
*MRPS18B*	mitochondrial ribosomal protein S18B	-0,81	1,63E-02	Male
*NIFK*	nucleolar protein interacting with the FHA domain of MKI67	-0,91	4,04E-02	Male
*PSMA4*	proteasome subunit alpha 4	-0,62	4,04E-02	Male
*SPCS3*	signal peptidase complex subunit 3	−1,24	4,45E − 03	Male
*SSB*	Sjogren syndrome antigen B	−0,75	1,01E-02	Male
*TMED7*	transmembrane p24 trafficking protein 7	-0,75	4,29E-02	Male
*UFM1*	ubiquitin fold modifier 1	−1,17	4,45E-03	Male
*CYP2D6*	cytochrome P450 2D14	1,39	3,27E-02	Male

*L2FC* log2 fold change, *p-adj p* value adjusted (BH correction)

#### Muscle

A total of 11.890 genes were included in DESEQ2 to perform differential expression analysis. We identified four interaction genes in muscle; of these three were down regulated [myosin light chain kinase family member 4 (*MYLK4),* interferon-induced very large GTPase 1-like *(GVINP1), novel Mt. t-RNA*)], while STAM binding protein like 1 (*STAMBPL1)* was up regulated (Table 2).

**Table 2:** Interaction genes and DE genes in female in muscle tissue (p-adj < 0.05)

Symbol	Gene description	L2FC	p-adj	
*MYLK4*	myosin light chain kinase family member 4	−2,27	4,53E-02	Interaction
*–*	novel Mt. tRNA	−3,16	2,65E-03	Interaction
*STAMBP1*	STAM binding protein like 1	1,47	4,53E-02	Interaction
*GVINP1*	interferon-induced very large GTPase 1-like	−1,56	2,14E-03	Interaction
–	novel Mt. tRNA	2,54	9,13E-05	Female

*L2FC* log2 fold change, *p-adj p* value adjusted (BH correction)

### GSEA functional enrichment analysis

#### Liver

Gene set enrichment analysis with GSEA was performed using the entire set of genes included in the DE analysis.

In males, we identified a total of thirteen statistically significant *Kyoto Encyclopaedia of Genes and Genomes* (KEGG) pathways, while only 8 in females (Table 3). Interestingly, the genes belonging to the *proteasome* KEGG pathway were down regulated in EO in the male group (ES = − 0.37), and up regulated in the female group (ES = + 0.37). A gene to be mentioned is *PSMA4*. *PSMA4* belongs to the *proteasome KEGG* pathway and it is a statistically significant down regulated DE gene in males.

**Table 3:** Statistically significant GSEA summary results in liver of males and females (FDR < 0.05)

	KEGG pathway	Size	ES	NES	FDR
Males	Protein Export	15	−0.60	0.000	0.001
	Proteasome	33	−0.37	0.000	0.002
	Antigen Processing and Presentation	20	−0.43	0.000	0.006
	Rna Degradation	16	−0.38	0.012	0.116
	Aminoacyl Trna Biosynthesis	19	−0.30	0.031	0.310
	Ppar Signaling Pathway	29	0.43	0.000	0.000
	Glycolysis Gluconeogenesis	21	0.48	0.000	0.001
	Drug Metabolism Cytochrome P450	20	0.44	0.000	0.006
	Glycine Serine and Threonine Metabolism	22	0.38	0.002	0.034
	Peroxisome	48	0.26	0.000	0.028
	Retinol Metabolism	16	0.43	0.006	0.031
	Citrate Cycle Tca Cycle	20	0.37	0.002	0.037
	Metabolism Of Xenobiotics By Cytochrome P450	17	0.41	0.006	0.033
Females	Cytokine Cytokine Receptor Interaction	94	−0.21	0.000	0.019
	Pathogenic *Escherichia coli* Infection	35	0.40	0.000	0.001
	Proteasome	34	0.37	0.000	0.003
	Amino Sugar and Nucleotide Sugar Metabolism	36	0.33	0.000	0.009
	Glycolysis Gluconeogenesis	33	0.34	0.000	0.017
	Ecm Receptor Interaction	55	0.25	0.000	0.022
	Cysteine and Methionine Metabolism	25	0.35	0.004	0.037
	Protein Export	19	0.39	0.002	0.047

Size = Number of genes. *ES* Enrichment score. ES represents the overepresentation degree of top and bottom genes included in ranked list based on log2FoldChange of DE genes included. *NES* Normalized enrichment score

#### Muscle

GSEA identified sixteen significant KEGG pathways in males and twenty-six in females. Six pathways were identified in both sexes (*Proteasome, Spliceosome, Ribosome, Pyrimidine Metabolism, Peroxisome, Hematopoietic Cell Lineage*) (Table 4).

**Table 4:** Statistically significant GSEA summary results in muscle of males and females (FDR < 0.05)

	KEGG pathway	Size	ES	NES	FDR
Males	Proteasome	34	−0.47	−3.14	0.000
	Ecm Receptor Interaction	58	−0.35	−3.11	0.000
	Complement and Coagulation Cascades	30	−0.41	−2.72	0.001
	Spliceosome	95	−0.21	−2.32	0.012
	Ribosome	36	−0.32	−2.31	0.009
	Pyrimidine Metabolism	62	−0.24	−2.20	0.016
	Antigen Processing and Presentation	32	−0.32	−2.15	0.020
	Ppar Signaling Pathway	44	−0.26	−2.06	0.031
	Rna Degradation	48	−0.25	−2.03	0.034
	Peroxisome	60	−0.21	−1.96	0.046
	Hematopoietic Cell Lineage	40	−0.27	−1.96	0.043
	Ubiquitin Mediated Proteolysis	102	0.21	2.46	0.012
	Basal Cell Carcinoma	29	0.37	2.39	0.012
	Notch Signaling Pathway	37	0.33	2.33	0.015
	Mapk Signaling Pathway	177	0.15	2.31	0.013
	Wnt Signaling Pathway	96	0.19	2.18	0.025
Females	Peroxisome	60	−0.30	−2.77	0.000
	Fatty Acid Metabolism	28	−0.41	−2.62	0.001
	Spliceosome	95	−0.23	−2.61	0.002
	Butanoate Metabolism	20	−0.47	−2.53	0.002
	Proteasome	34	−0.36	−2.52	0.002
	Pyrimidine Metabolism	62	−0.24	−2.23	0.011
	Ribosome	36	−0.29	−2.09	0.025
	Propanoate Metabolism	23	−0.36	−2.00	0.038
	Valine Leucine and Isoleucine Degradation	35	−0.28	−1.96	0.043
	Nucleotide Excision Repair	37	−0.27	−1.93	0.045
	Lysine Degradation	33	−0.28	−1.93	0.042
	Tryptophan Metabolism	21	−0.34	−1.91	0.043
	Ecm Receptor Interaction	58	0.45	4.03	0.000
	Complement and Coagulation Cascades	30	0.56	3.67	0.000
	Focal Adhesion	159	0.20	2.89	0.000
	Hematopoietic Cell Lineage	40	0.37	2.77	0.000
	Regulation Of Actin Cytoskeleton	148	0.19	2.71	0.000
	Lysosome	93	0.23	2.64	0.000
	Pathogenic Escherichia Coli Infection	35	0.34	2.44	0.003
	Cell Adhesion Molecules Cams	57	0.26	2.31	0.006
	Endocytosis	128	0.17	2.30	0.006
	Sphingolipid Metabolism	23	0.38	2.21	0.011
	Cytokine Cytokine Receptor Interaction	88	0.20	2.16	0.015
	Pentose Phosphate Pathway	19	0.40	2.04	0.030
	Leukocyte Transendothelial Migration	74	0.19	1.93	0.051
	Glycolysis Gluconeogenesis	32	0.28	1.93	0.048

Size = Number of genes; *ES* Enrichment score. ES represents the overepresentation degree of top and bottom genes included in ranked list based on log2FoldChange of DE genes included; *NES* Normalized enrichment score

### WGCNA and enrichment functional analysis

#### Liver

WGCNA was used to generate co-expression networks using the male and female datasets separately in order to identify modules containing genes affected by EO diet that might be part of the same biological pathway.

The diet was used as the trait in order to construct module-trait relationship and a total of 38 merged modules (MMs) were identified in males. Among these, three were significant (*p*-value < 0.05) modules, which were tightly correlated to the EO diet in the male liver tissue: the Male_Module5 (cor = + 0.76, *p*-value = 0.03), the Male_Module6 (cor = + 0.82, *p*-value = 0.01) and the Male_Module36 (cor = − 0.72, *p*-value = 0.04).

Genes included in each significant module (59 in Male_Module5, 110 in Male_Module6 and 48 in Male_Module36) were uploaded into ClueGO to identify significant cellular components (CC), biological processes (BP), molecular function (MF) and KEGG pathways (FDR < 0.05). No significant GO categories were found for Male_Module5.

The Male_Module6 was the most correlated module to EO diet. Among the 20 BP identified by ClueGO, we found that genes included in Male_Module6 were enriched for GO terms related to response to stimuli and regulation of immune response: GO:0090287-regulation of cellular response to growth factor stimulus; GO:0071560-cellular response to transforming growth factor beta stimulus; GO:0090288-negative regulation of cellular response to growth factor stimulus; GO:1903844-regulation of cellular response to transforming growth factor beta stimulus; GO:2001020-regulation of response to DNA damage stimulus; GO:2001022-positive regulation of response to DNA damage stimulus; GO:0002821-positive regulation of adaptive immune response; GO:0050864-regulation of B cell activation; GO:0030217-T cell differentiation; GO:0002821-positive regulation of adaptive immune response. Also, genes included in the Male_Module6 were involved in RNA degradation pathway (ko:03018) (see Additional file 1)*.*

Furthermore, the genes included in Male_Module36 were enriched for 6 GO terms: GO:0004857-enzyme inhibitor activity, GO:0031023- microtubule organizing center organization, GO:0031300- intrinsic component of organelle membrane, GO:0036064-ciliary basal body, GO:0051222- positive regulation of protein transport, GO:0090288-negative regulation of cellular response to growth factor stimulus (Additional file 2).

In the female dataset, we identified 36 modules, and two of them were statistically significant MMs: the Female_Module36, which included 133 genes (cor = − 0.8, *p*-value = 0.02) and the Female_Module16, which included 363 genes (cor = + 0.7, *p*-value = 0.05).

The results obtained by ClueGO showed that the 363 genes included in the Female_Module16 module were enriched for 20 BP, 1 CC and 2 MF. Interestingly, among these genes we found *FGG* and *FGB,* which represent interaction genes identified in the DE analysis. These genes were overrepresented in blood coagulation, fibrinolysis, platelet aggregation and apoptotic signalling related GO terms (see Additional file 3).

The Female_Module16 was the biggest module identified in the liver tissue and in view of this we looked into intramodular attributes: *module-membership* and *gene significance.*

We selected the genes with *module-membership* higher than 0.9. These genes were then used in ClueGO for enrichment analysis. Twenty-one GO terms related to purine nucleotide biosynthetic processes, mitophagy, regulations of mRNA processing and macromolecular glycosylation were statistically significant exclusively in the Female_Module16. Also, we found significant enrichment for the KEGG pathway (ko:04141- Protein processing in endoplasmic reticulum) (see Additional file 4).

#### Muscle

In analysis of the muscle tissue in the males, we identified a total of 55 modules. Among these, four were significantly correlated to EO diet: the Male_Module52 included 54 genes (cor = + 0.72, *p*-value = 0.04); the Male_Module25 included 47 genes (cor = − 0.76, *p*-value = 0.03); the Male_Module27 included 48 genes (cor = − 0.74, *p*-value = 0.04) and the Male_Module38 included 188 genes (cor = + 0.80, *p*-value = 0.02).

The entire lists of genes in each module were further analysed using ClueGO plugin.

Male_Module38 was strongly correlated to EO diet. Genes included in Male_Module 38 were enriched for29 significant GO-Terms and 4 KEGG pathways (FDR < 0.05). The enrichment analysis revealed that EO diet influenced numerous pathways, such as regulation of protein signal transduction, NF-kappa B-signalling pathway and pyrimidine metabolism (see Additional file 5). The Male_Module52 was enriched for GO terms related to ion transport: GO:0022853-active ion transmembrane transporter activity; GO:0072511-divalent inorganic cation transport; GO:0070838-divalent metal ion transport; GO:0006816-calcium ion transport (see Additional file 6).

No significant GO terms were identified for the Male_Module25 and Male_Module27 modules.

In the analysis of muscle tissue in the females, 57 modules were identified and only one was associated to the EO diet: Female_Module13 (cor = − 0.73, *p*-value = 0.04). We found no GO terms associated to this module.

### Module comparison between sexes in both tissues

The DE genes revealed a different response to EO diet supplementation in males vs. females. Therefore we compared male and female co-expression networks for both tissues. We applied a Fisher test to compute the overlap of modules between sexes.

**Fig. 1:**
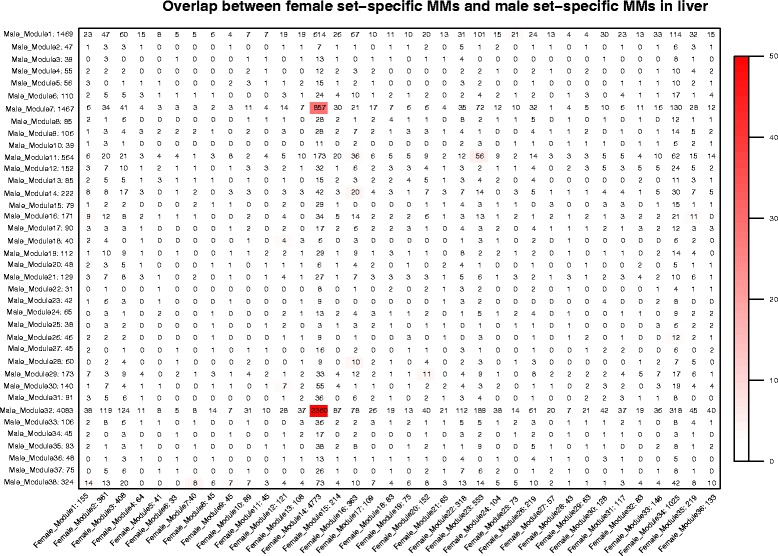
Overlap between female set-specific MMs and male set-specific MMs in liver. The overlap between all modules identified in males and in females is represented in the table. Each row in the table corresponding to one male set-specific module and the column corresponds to one female set-specific module. The modules with the same number did not represent the same type of co-expressed genes included (Male_Module1 ≠ Female_Module1). The number in the table indicates the genes counted in the intersection between each module identified in both sexes. Colouring of the table indicates significant overlap evaluated using Fisher’s exact test. The intensity of the colour is proportional to the Fisher’s exact test *p*-value for the overlap of the two modules. The stronger red colour represents the more significant overlap

In liver the modules highly correlated to EO in males (Male_Module5, Male_Module6, Male_Module36), with *p*-value < 0.05 did not have counterpart modules in female (Fig. 1). The coloured boxes in Fig. 1 represented the genes counted in the overlap of the corresponding modules, which were no statistically correlated to EO diet (*p*-value > 0.05). As in liver, in muscle highly the modules correlated to EO (*p*-value < 0.05) in male did not have a counterpart in female.

### Consensus analysis

We generated a consensus network to identify conserved modules correlated to EO diet in males and females in each tissue.

Among the 37 consensus modules (consMs) identified in liver, only ConsM_K was significant in both sexes (*p*-value < 0.05). ClueGO analysis revealed that genes included in ConsM_K were enriched for 73 BP, 10 CC, 3 MF and 3 KEGG pathways. Interestingly, all BP, 8 CC, 2 MF and 2 KEGG pathway (ko:04610 - Complement and coagulation cascades; ko:04611- Platelet activation) included two of our DE genes: *FGG* and *FBG*, which were mentioned previously. Specific details of ConsM_K and associated enrichment analysis results are presented in Additional file 7.

We also identified 76 consMs in muscle. None of them were significant in both males and females (*p*-value< 0.05).

### Regulatory genes investigation

The ConsM_K module, identified in liver, was further analysed with the Lemon-Tree algorithm to investigate potential regulator genes. In the males we found two regulatory genes involved in immune and inflammation pathways: catenin alpha like 1 (*CTNNAL1)* and nuclear transcription factor, X-box binding 1 (*NFX1)*.

## Discussion

Liver and muscle are important target tissues in nutrigenomics investigation, taking into account that liver has a central role in numerous metabolic processes [36], while muscle (meat quantity and quality) is an important economic trait and is related to lamb growth [12, 37]. For this reason, using an RNA Seq-approach we analysed the transcriptome of 32 samples of liver and muscle tissues of males and females lambs to evaluate which genes were influenced by EO diet, considering the interaction with sex. This is the first nutrigenomics investigation in lambs fed with EOs where whole transcriptomic analyses have been performed. Our study suggests sex dependent effect of the diet on the gene expression profile of both tissues. The functional analysis reveals that the EO diet affected mainly the inflammatory and the immune related pathways. Here, we discuss the main evidences that support the hypothesis of a sex-dependent effect of the diet in liver and in muscle tissue.

### Effect of EO treatment in liver

In the DE analysis, we observed a sex-dependent effect of the diet, modulated by the sex, influenced the expression of genes encoding heat shock proteins – *DNAJB9* and *HSP90B1* [38, 39] – and the expression of genes involved in primary homeostasis, ER stress response and inflammatory pathways: *FGG* and *FGB* [40, 41], *UFM1* [42, 43], *HP* [44], *KDERL2* [45], *MANF* [46]. Interestingly, our interaction genes, *HP* and *MANF,* are genes influenced by diet treatment in cows. The expression of *HP*, a *NF-kB* target gene, is decreased in dairy cow fed with essential oil supplementation [44]; while *MANF* is up-regulated in the liver cells of cattle under dietary restriction and the high expression is associated with inhibition of hepatic cellular proliferation [47].

The use of diet * sex model allowed to observe a different response to the diet treatment in males and in females, with the greater effect of EO diet found in the male lambs. Fifteen DE genes were found in males; of these, fourteen were down regulated in EO (*FGB, FGG, DNAJB9, HSP90B1, PSMA4, KDELR2, SSB, CALM2, SPCS3, UFM1, MRPS18B, NIFK, ARG1, TMED7,*) and, among the down regulated DE genes, almost half of them – *FGB, FGG, DNAJB9, HSP90B1, PSMA4, KDELR2* – were mainly involved in inflammatory, immune and stress responses. *FGB* and *FGG* are exclusively expressed in liver cells and play a role in primary haemostasis, as components of blood clots [40]. The transcription of these genes is influenced by acute-phase inflammatory response (APR) and IL-6 during the inflammation process [40]. *DNAJB9* (*HSP40*) and *HSP90B1* genes encode important heat shock proteins (Hsps) and they were down regulated in EO in males. These genes play a role in stress response during infection, and inflammation conditions, where the expression of Hsps is higher [48]. *DNAJB1* belongs to HSP40 family plays a role in inflammatory conditions and autoimmune diseases [39]. HSP90b1 protein is involved in the maintaining of the ER proteins homeostasis, acting on ER unfolded-protein response (UPR) [38, 49]. Also, HSP90b1 has implicated in assembly of immunoglobulin [38]. Other down-regulated DE genes in males fed EOs were PSMA4, a component of proteasome, which is involved in the major histocompatibility complex-class I antigen processing [50] and, *KDELR2* that plays an important role in immunotoxin pathway [45]. EOs act in modulating a wide number of cellular responses in animals [51] and our results provide evidence that EOs modulated anti-inflammatory and stress response related genes. The lower expression of *FGB, FGG, DNAJB9, HSP90B1, PSMA4, KDELR2* genes in males show that dietary EOs could be an interesting strategy to preserve lamb wellness, given the essential oil anti-inflammatory proprieties [11] and that EO supplementation in lamb could be used not only for its direct beneficial effect on gastrointestinal trait [14, 15, 19, 21].

In addition, GSEA analysis of differential expression profiles and the ClueGO functional analysis of liver modules identified in WGCNA provided evidence of the effect of EO diet on inflammatory and immune pathway. In males, the WGCNA module with the highest correlation value to EO (Male_Module6) included a cluster of co-expressed genes overrepresented by *positive regulation of adaptive immune response*, *regulation of B cell activation, T cell differentiation and positive regulation of adaptive immune response* related GO terms*.*

Moreover, WGCNA in liver revealed three hub genes – *DNAJB9, MANF* and *UFM1* – strongly correlated with the EO diet*.* Interestingly, they were included in the biggest module identified in females (Female_Module16)*.* However, *MANF, DNAJB9,* and *UFM1* represented interaction genes in DE analysis and *DNAJB9, UFM1* were also down regulated DE genes in males and this provided evidence that these genes were significantly influenced by the EO diet. Besides, the involvement of *DNAJB9* in stress response and the high expression of *UFM1* in diseases associated to ER stress [42, 43] showed that EO supplementation could have potential beneficial effects.

The use of WGCNA provided new evidences for the association between EO treatment and the genes involved in immune response, which were overrepresented by immune response related GO terms*.* Also, WGCNA analysis confirmed that *DNAJB9, MANF* and *UFM1* were the more affected by the diet.

### Effect of EO treatment in muscle

In contrast to the liver results, in muscle the EO seems to have a minor effect. Indeed, only four genes affected by the sex treatment interaction were identified in DE analysis (*MYLK4,* GVINP1*, novel Mt-tRNA, STAMBPL1*) and, only one DE gene (*novel Mt-tRNA*) discriminated the different response to EO diet in males vs. females. The different effect of EO diet on muscle compared to liver could be related to the tissue function: liver represents an important interface between diet and the metabolic process. Furthermore, the liver plays a crucial role in distributing nutrient to the peripheral tissues, such as the muscle [36].

GSEA results revealed that the *Regulation of actin cytoskeleton* KEGG pathway, which was comprised of up regulated genes in EO including the *MYLK* genes (*MYLK* and *MYLK3*), was enriched only in females. The *MYLK* genes have an important role during smooth muscle contraction [52] and in development of actomyosin filaments [53], and it is associated to meat quality [54]. Interestingly, in Malila et al. [54] *MYLK2* is included in *Actin cytoskeleton signalling* pathway, associated to the pale, soft, and exudative (PSE) meat defect. Compared to the normal meat, *MYLK2* is down regulated in PSE turkey meat [54]. In view of this, we can speculate that the EO diet improve the meat quality in female lambs, increasing the expression of *MYLK* genes. Therefore further analyses on the effects of the EO diet on *MYLK* genes expression related to lamb meat quality could be interesting, taking into account that our EO diet supplementation prove to be effective on meat oxidative stability in lamb (Trabalza-Marinucci, unpublished data).

### Comparison between sexes in both tissues: Sex-dependent effect of the diet

The DE and WGCNA analyses supported our hypothesis that EO had a sex-dependent effect, thus we compared male and female co-expression networks built through WGCNA in both tissues. As expected, the comparison in liver resulted in a low number of intersecting genes between modules and showed the low similarity between modules obtained in males vs. females. However, the same results were found in muscle, suggesting that the EO diet influenced different co-expressed genes in liver as well, even though the DE analysis in muscle did not reveal a significant EO diet-sex dependent effect.

Interestingly, we confirmed the EO diet-sex dependent effect in liver using consensus networks. Through this approach we identified conserved modules both in males and females, including the same co-expressed genes correlated to EO diet. Noteworthy, the consensus module ConsM_K – included co-expressed genes in opposite correlation to the EO in the two sexes (cor = − 0.78 in males and cor = + 0.81 in females). Since FGB and FGG genes have been found among ConsM_K co-expressed genes, WGCNA provided itself useful in obtaining further evidences on how EOs influenced the expression of the former genes in an opposite way in males and females.

In the key consensus module (consM_K) we identified two regulatory genes *CTNNAL1* and *NFX*. Both genes were associated with inflammatory and immune pathways: The CTNNAL1 protein increases the NF-κB pathway activity, which modulates immune response and apoptosis in inflammatory states [55]. NFX1 was identified as a class II major histocompatibility complex (MHC) inhibitor [56], but recent discoveries show that different NFX1 isoforms regulate telomerase (hTERT) expression, influencing NF-κB pathway activity during papilloma virus infection in humans [57, 58]. The identification of *CTNNLA1* and *NFX1* as regulatory genes related to immune and inflammatory pathway in our analysis showed that EO diet had a relevant impact in these biological processes in the liver of male lambs, according to DESEQ2 and enrichment analyses of WGCNA modules.

Genomic, transcriptomic and metabolic analysis reported sexual dimorphism in metabolic tissues and metabolite concentration profiles [59–61]. Moreover, sex-dependent activation of biological pathways in response to a specific diet has been previously described. For instance, milk-based diets influence liver enzymes quantity in males rats, while fish-based diets have a stronger effect on liver fatty acid composition in females, showing that basal differences in liver lipid composition between male and females are modulated by diet [62]. Moreover, in vitro studies reveal a sex-specific drug metabolism in the liver of goats [63] and nutritional challenges can influence sex-specific metabolic modifications in adult sheep [64].

A sex-specific effect was also observed in our study in the lamb transcriptome. A possible explanation for this effect might be a potential different biotransformation of the essential oils in the two sexes, taking into account that in both cattle and pigs a sex-specific activation of several metabolic pathways has been reported [59, 61].

In our findings we observed a stronger EO diet effect in male liver. Given the down regulation of genes mainly involved in inflammatory and stress response, and based on previous results showing EOs anti-inflammatory proprieties [11, 65, 66], the authors can speculate that EOs might have a more pronounced effect on male individuals improving the metabolic and functional status of liver.

## Conclusion

Our study revealed for the first time a sex-dependent effect of dietary EOs on the transcriptome in lambs. A stronger EO effect was observed in liver of male lambs that seems to affect the immune response related pathways, suggesting that EO-enriched diets could have a beneficial effect on lambs’ health. However, this hypothesis could be verified challenging the animals with a stressful environment (e.g heat, farming system).

In this way, our findings expand the knowledge of the use of EO as a nutritional strategy, using lamb as a model for nutrigenomics investigations, and provide useful pieces of information to be considered in the experimental design of future similar studies. These findings could be also considered for sex specific strategy in livestock and in human diets. This study can provide a basis for a follow-up study in a larger population using qPCR to validate the genes identified.

## Methods

### Experimental design

**Fig. 2:**
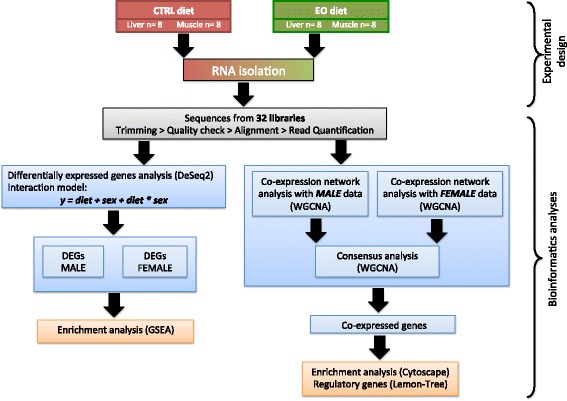
Experimental design and data analysis workflow

The workflow used in our study is shown in Fig. 2.

In this study we used 16 *Appenninica × Sarda* lambs (175 ± 10 day-old), with a mean body weight of 32.2 ± 4.1 kg at the beginning of the trial (Body weight_begin_), obtained from the didactic farm of the Department of Veterinary Medicine, University of Perugia.

The 16 animals used in the study were divided in two homogeneous experimental groups, which included four males and four females each. One group was fed with a control diet (CTRL), based on alfalfa hay (1.5 kg) and a commercial concentrate feed (270 g), while the other was fed the CTRL diet supplemented with three ml of a 1:1:1 mix of cinnamon bark, eucalyptus leaves and dill seed essential oils (EO). The essential oil composition is detailed in [21]. The mix of essential oils was absorbed by a matrix of β-cyclodextrins (30 g) and thoroughly mixed with the concentrate before feeding, in order to make it easier for the lambs to consume the product. The CTRL lambs received the same amount of cyclodextrins without essential oils.

The lambs were kept in separated pens with straw bedding equipped with identical mangers and had free access to water.

The experiment was conducted for 30 days and the diet was administrated twice a day in equal parts. At the end of the trial, the body weight of all lambs (Body weight_end_) was measured. No statistically significant difference between Body weight_end_ and Body weight_begin_ was found. In addition the same values of feed intake for each lamb were observed.

### RNA extraction

At slaughtering, liver and muscle tissue samples were collected from all lambs. Tissues were frozen in liquid nitrogen and stored at − 80 °C.

Total RNA from all samples was extracted according to the TRIzol Plus RNA Purification Kit manufacturer specifications (Ambion – Life Technologies, MA, USA) from about 100 mg for muscle and 50 mg for liver, powdered with liquid nitrogen, mortar and pestel. To remove genomic DNA from samples we used DNAse treatment according to the TURBO DNAse manufacturer specifications (Ambion – Life Technologies). To inactivate the DNAse, RNA from each sample was purified using phenol-chloroform-isoamyl alcohol method, according to Sambrook et al. protocol [67].

RNA quantity and quality were evaluated using NanoDrop 2000 spectrophotometer (Thermo Fisher Scientific, Waltham, MA, USA) and Qubit 2.0 Flurometer (Life Technologies, MA, USA), while RNA integrity was assessed by microfluidic electrophoresis on BioAnalyzer 2100 (Agilent Technologies).

### RNA sequencing

RNA-seq, libraries were prepared according to the Illumina TruSeq2 kit, using poly-A mRNA purification.

A total of 32 RNA samples, 16 for muscle and for liver, respectively, were sequenced on two lanes of Illumina HiSeq 1500 platform generating 101 base-paired end reads.

The quality of raw sequences was checked using FastQC (http://www.bioinformatics.babraham.ac.uk/projects/fastqc/). The adapter removal and trimming were done with Trimmomatic v.0.33 [68].

Afterwards, high quality reads were aligned using STAR v.2.4.0.1 [69] to the sheep Ensembl reference genome (*Ovis aries* v.3.1) setting the other parameters to STAR default values. HTSeq v.0.6.1 [70] was used to quantify the mapping of the reads at each gene locus based on genomic features annotated in Ensembl *Ovis aries* (v.3.1).

### Differential expression analysis

We filtered genes with low counts in both tissues. Only genes with at least one count-per-million (CPM) in more than 50% of the samples were included in the following analyses (liver = 11.773 genes; muscle = 11.890 genes).

Differentially expressed (DE) genes in liver and muscle were investigated using DESEQ2 R package (v 1.12.3) [71].

DESEQ2 fits a generalized linear model assuming a negative binomial distribution of the read counts. We fitted the following model:

$$ y= diet+ sex+ diet\ast sex $$

Where y is the gene expression counts, diet is the factor distinguishing between treatments (CTRL vs. EO diet), sex is the factor distinguishing between male and female animals and diet*sex is the interaction between the treatment and sex and. The model evaluates the effect on gene expression of 1) the treatment (CTRL vs. EO); 2) the sex (Males vs. Females); and 3) the interaction between treatment and sex.

We considered genes to be DE when the significance level (adjusted *p*-value for multiple comparisons) was lower than 0.05, after Benjamini-Hochberg correction (padj).

### Sex-specific weighted gene co-expression network analysis (WGCNA)

Differential expression analysis revealed a significant interaction between treatment and sex, meaning a different diet response between males and females in both tissues. Therefore we performed co-expression analysis separately for the two sexes. Male and female expression data was transformed using *varianceStabilizingTransformation* function in DESEQ2 R package [71].

Sex-specific co-expression networks were built using WGCNA [34]. Briefly, pairwise Pearson’s correlation matrix of expression values was transformed into an adjacency matrix using a power function to ensure a scale free topology of the network [72]. WGCNA assumes a scale-free topology of the co-expression network. A power of *Beta* was chosen in the interval (1–15), to ensure a scale-free topology of the network with scale-free topology index (R^2^) > 0.80.

In the male and female liver data we estimated a β value of 6, while in male and female muscle data we estimated a β value of 9.

The adjacency matrix was used to calculate the Topological Overlap Measure (TOM) corresponding to the overlap between pairs of interconnected genes [72].

TOM was used to build a gene clustering dendrogram. Highly interconnected genes were clustered by using the *dynamicTreeCut* algorithm [72] with all the parameters at default values. Each cluster also called “module” was arbitrarily labelled with a unique colour (e.g brown4).

Next, for each module a module eigengene (ME) was calculated. The ME is a measure of the representative expression profile of all genes included in each module [72]. Highly interconnected modules were merged using the *mergeCloseModules* function [72], based on the MEs correlation. The merged modules were used in the rest of analysis. The Module-trait relationship (MTR) was computed as the Pearson correlation between MEs and the diet. Modules with MTR correlation *p*-value < 0.05 were further analysed.

#### Consensus WGCNA for identifying conserved gene modules

We generated a consensus network to identify conserved modules among males and females. More specifically, using male and female independent datasets, WGCNA Consensus analysis evaluated which are the modules present in both data sets. For each tissue, a consensus TOM was generated keeping the minimum value between the two TOM matrices described above (male and female). The consensus TOM was used to identify the modules and highly correlated modules were merged following the same procedure used for the sex specific network. The resulting Consensus modules (consMs) were used in the rest of the analysis. We evaluated the consensus MTR for each network (males, females and consensus) in both tissues.

To facilitate reading, each colour name has been arbitrarily replaced, thus for instance brown4 has named Module1. To discriminate sex-specific identified module, suffixes Male_ and Female_ have been specified, for instance Male_Module1 and Female_Module1. The modules with the same number did not represent the same type of co-expressed genes included.

Upper and lowercase letters have been used to define Consensus modules name, for instance consM Darkgrey has been renamed as consM_K.

### Functional enrichment analysis

Enrichment analysis of DE genes was performed for both tissues using Gene Set Enrichment Analysis (GSEA) based on *Kyoto Encyclopaedia of Genes and Genomes* (KEGG) pathways gene set collections from human Molecular Signatures Database (MSigDB) [73].

The analysis included all the genes used in the DE analysis after the DESEQ2 independent filtering procedure (N°genes _liver_ = 11.771; N°genes _muscle_ = 11.886). The log2 fold change (L2FC) was used to rank the genes.

Genes from significant modules identified in the WGCNA analysis were used for functional enrichment analysis using ClueGO v.2.5.0 with a Cytoscape v3.4.0 plugin [74]. The ovine Entrez gene IDs from each module was used as input and all ovine Entrez gene IDs included in the co-expression analysis was used as Custom Reference Set.

Significance threshold for KEGG pathway and GO terms was set at *p*-value < 0.05, after Benjamin-Hochberg correction (FDR).

### Investigation of regulatory genes

Regulatory genes in consMs were detected using Lemon-Tree algorithm [75]. The list of potential regulator genes list (*N* = 2.510) was obtained taking into account of three GO categories, *Signal Transducer* (GO ID: 0007165), *Signal Transducer activity* (GO ID: 0004871) and *Kinase activity* (GO ID: 0016301). The analysis was performed using a centered and scaled male and female gene expression matrix, including the list of genes identified in module from consensus WGCNA.

## Additional files

Additional file 1: **Table S1.** Liver Male_Module6. Significant GO terms and KEGG pathways by ClueGO enrichment analysis (FDR < 0.05). Information contained in the table are significant GO-ID, GO term accession number (. GO:0003018); GOTerm, name of GO term (e.g. RNA degradation); Ontology source, ontology vocabularies or Kyoto Encyclopaedia of Genes and Genomes (e.g. GO_BiologicalProcess or KEGG); FDR, False Discovery Rate after Benjamini-Hochberg correction (e.g. 20,0E-3); % Associated Genes, the percentage of input genes found per term (e.g. 6,90); Nr. Genes, number of input genes found per term (e.g. 4,00); Associated Genes Found, associated name of genes found per term (e.g. [EXOSC6, HSPA9, LSM1, SKIV2L2]). (XLS 31 kb)

Additional file 2: **Table S2.** Liver Male_Module36. Significant GO terms pathways by ClueGO enrichment analysis (FDR < 0.05). Information contained in the table are significant GO-ID, GO term accession number (e.g. GO:0004857); GOTerm, name of GO term (e.g. enzyme inhibitor activity); Ontology source, ontology vocabularies or Kyoto Encyclopaedia of Genes and Genomes (e.g. GO_MolecularFunction); FDR, False Discovery Rate after Benjamini-Hochberg correction (e.g. 47,0E-3); % Associated Genes, the percentage of input genes found per term (e.g. 4,65); Nr. Genes, number of input genes found per term (e.g. 4,00); Associated Genes Found, associated name of genes found per term (e.g. [DUS2, GLMN, HRG, OAZ2]). (XLS 28 kb)

Additional file 3: **Table S3.** Liver Female_Module16. Significant GO terms by ClueGO enrichment analysis (FDR < 0.05). Information contained in the table are significant GO-ID, GO term, accession number (e.g. GO:0042730); GOTerm, name of GO term (e.g. fibrinolysis); Ontology source, ontology vocabularies (e.g. GO_BiologicalProcess); FDR, False Discovery Rate after Benjamini-Hochberg correction (e.g. 18,0E-3); % Associated Genes, percentage of input genes found per term (e.g. 35,71); Nr. Genes, number of input genes found per term (e.g. 5,00); Associated Genes Found, associated name of genes found per term (e.g. [FGA, FGB, FGG, KLKB1, TMPRSS6]). (XLS 32 kb)

Additional file 4: **Table S4.** Enrichment analysis by ClueGo using highly connectivity genes in Female_Module16 module (FDR < 0.05). Information contained in the table are significant GO-ID, GO term, accession number (e.g. GO:0070085); GOTerm, name of GO term (e.g glycosylation); Ontology source, ontology vocabularies or Kyoto Encyclopaedia of Genes and Genomes (e.g. GO_BiologicalProcess or KEGG); FDR, False Discovery Rate after Benjamini-Hochberg correction (e.g. 16,0E-3); % Associated Genes, the percentage of input genes found per term (e.g. 4,65); Nr. Genes, number of input genes found per term (e.g. 4,00); Associated Genes Found, associated name of genes found per term (e.g. [B4GALT7, DDOST, DHDDS, DPAGT1]). (XLS 31 kb)

Additional file 5: **Table S5.** Liver Female_Module36. Significant GO terms by ClueGO enrichment analysis (FDR < 0.05). Information contained in the table are significant GO-ID, GO term, accession number (e.g. GO:0036064); GOTerm, name of GO term (e.g. ciliary basal body); Ontology source, ontology vocabularies (e.g. GO_CellularComponent); FDR, False Discovery Rate after Benjamini-Hochberg correction (e.g. 41,0E-3); % Associated Genes, the percentage of input genes found per term (e.g. 7,50); Nr. Genes, number of input genes found per term (e.g 3,00); Associated Genes Found, associated name of genes found per term (e.g. [CENPJ, KIAA0586, POC1A]). (XLS 34 kb)

Additional file 6: **Table S6.** Muscle male Blueviolet MM. Significant GO terms by ClueGO enrichment analysis (FDR < 0.05). Information contained in the table are significant GO-ID, GO term, accession number (e.g. GO:0006816); GOTerm, name of GO term (e.g. calcium ion transport); Ontology source, ontology vocabularies (e.g. GO_BiologicalProcess); FDR, False Discovery Rate after Benjamini-Hochberg correction (e.g. 610,0E-6); % Associated Genes, the percentage of input genes found per term (e.g. 4,94); Nr. Genes, number of input genes found per term (e.g 4,00); Associated Genes Found, associated name of genes found per term (e.g. [ATP2A3, CALCRL, MICU2, PML]). (XLS 28 kb)

Additional file 7: **Table S7.** Sheet 1 (ClueGO Results): Liver ConsM_K. Significant GO terms and KEGG pathway by ClueGO enrichment analysis (FDR < 0.05). Information contained in the table are significant GO-ID, GO term, accession number (e.g. GO:0007599); GOTerm, name of GO term (e.g. hemostasis); Ontology source, ontology vocabularies or Kyoto Encyclopaedia of Genes and Genomes (e.g. GO_BiologicalProcess or KEGG); FDR, False Discovery Rate after Benjamini-Hochberg correction (e.g. 39,0E-3); % Associated Genes, the percentage of input genes found per term (e.g. 4,35); Nr. Genes, number of input genes found per term (e.g. 3,00); Associated Genes Found, associated name of genes found per term (e.g [FGA, FGB, FGG]). Sheet 2 (Liver_consensus_module-trait): Liver consensus module-trait relationships. Information contained in the table are ConsMs, conserved modules in females and in males identified in Consensus analysis (e.g. consM_A); Cor, correlation value to EO diet (e.g. − 0.55) for females and males; *p*-value, corresponding *p*-value (e.g. 0.2). (XLS 55 kb)

## Abbreviations

BH: Benjamini-Hochberg correction
BP: Biological processes
CC: Cellular component
consM: Consensus module
CTRL: Control
DE: Differentially expressed
EO: Essential oil
FDR: False discovery rate
GO: Gene Ontology
GSEA: Gene set enrichment analysis
KEGG: Kyoto encyclopaedia of genes and genomes
L2FC: Log2 fold change
MF: Molecular Function
MM: Merged module
p-adj: *p* value adjusted
RNA-Seq: RNA-sequencing
WGCNA: Weighted gene correlation network analysis
